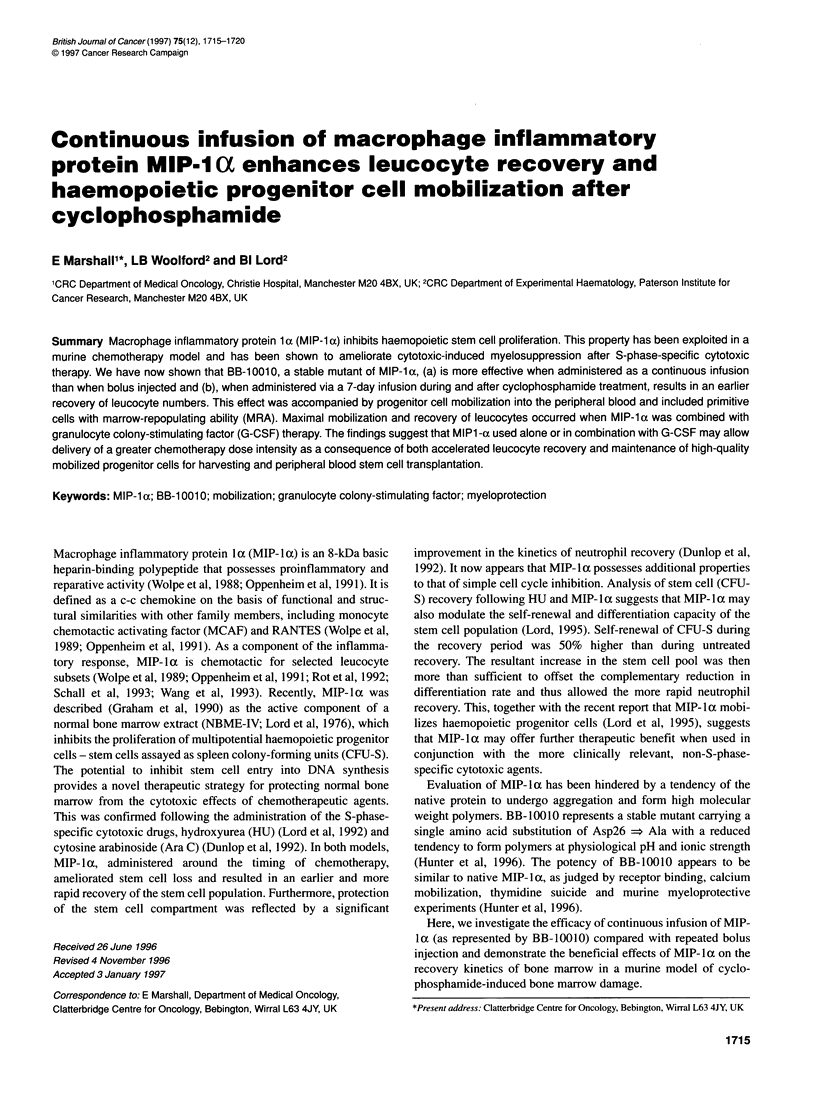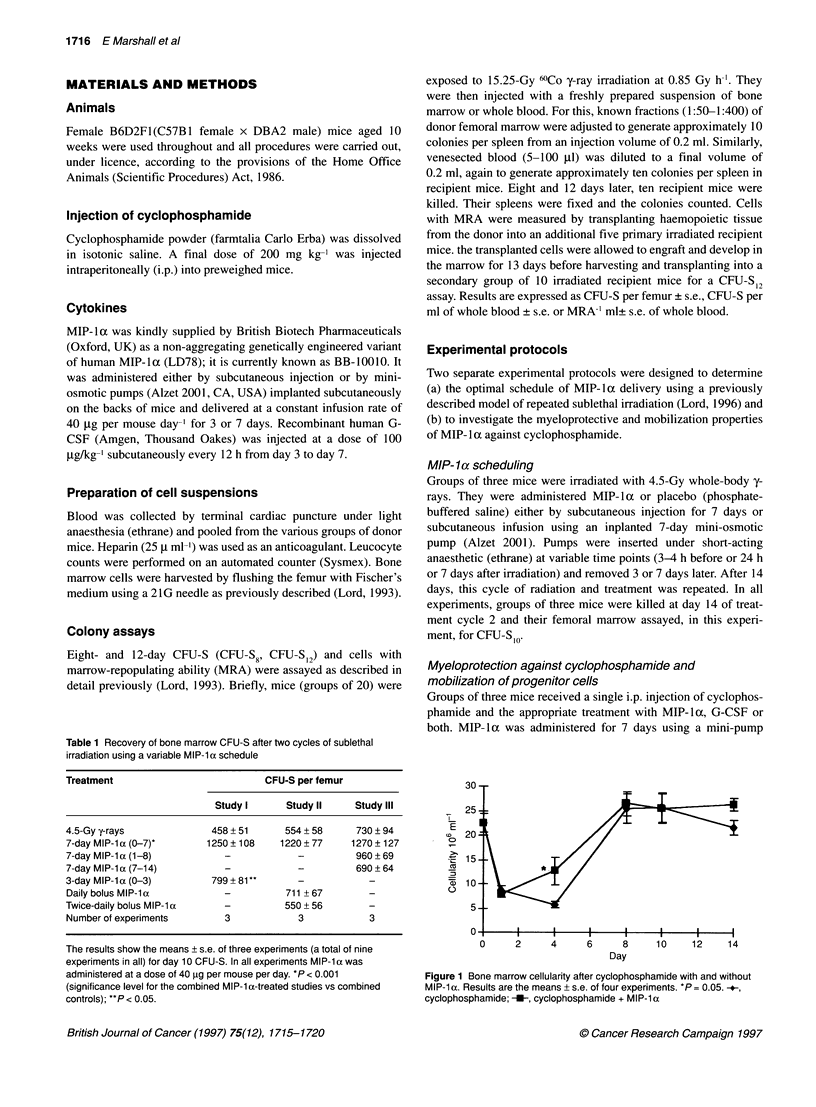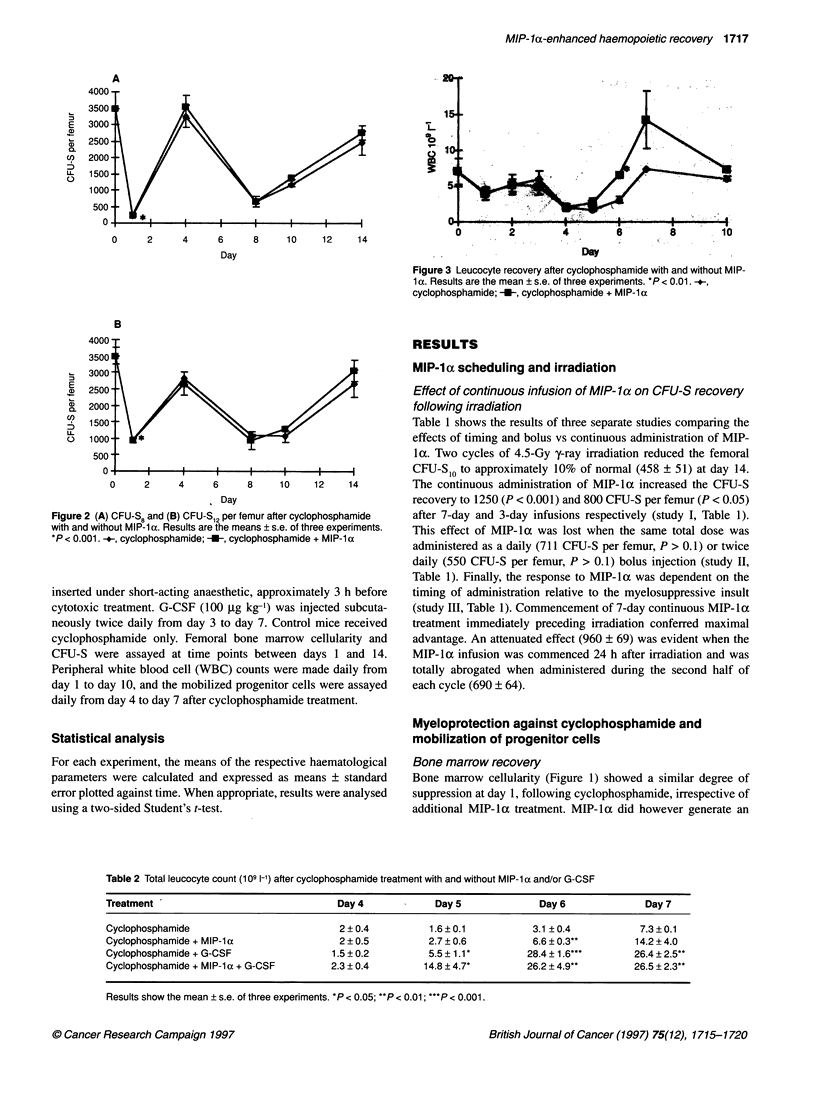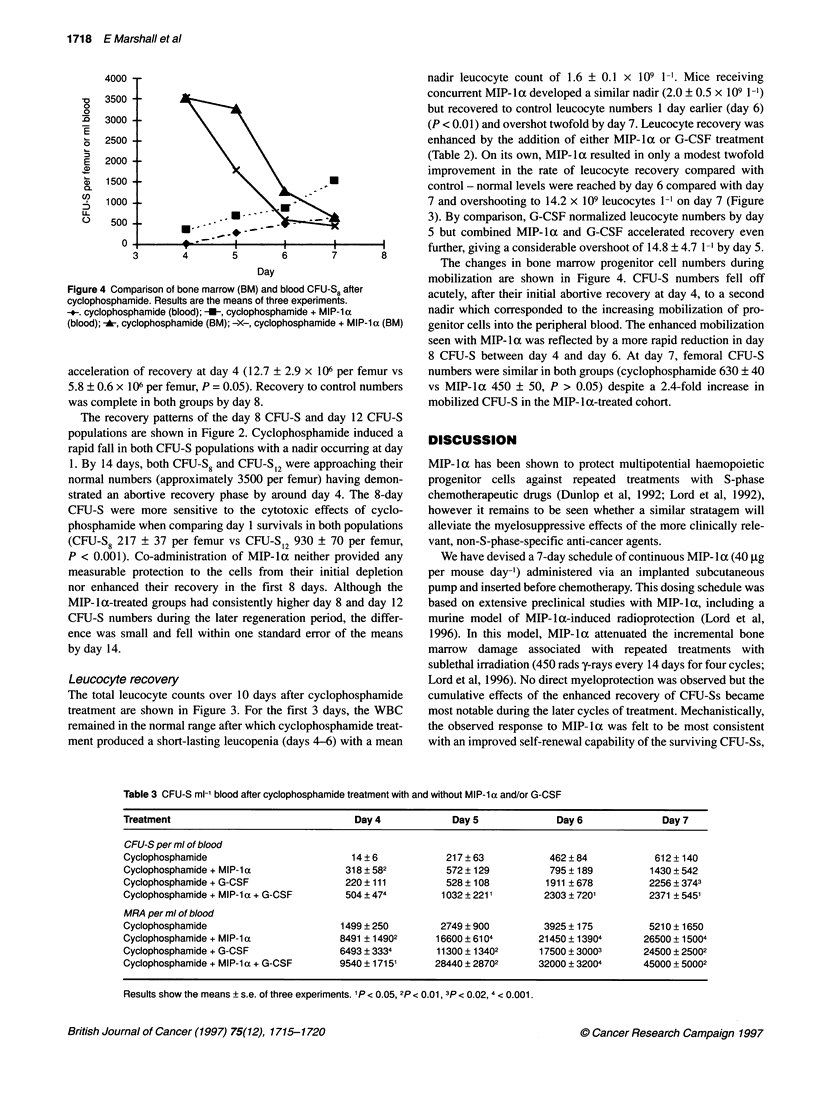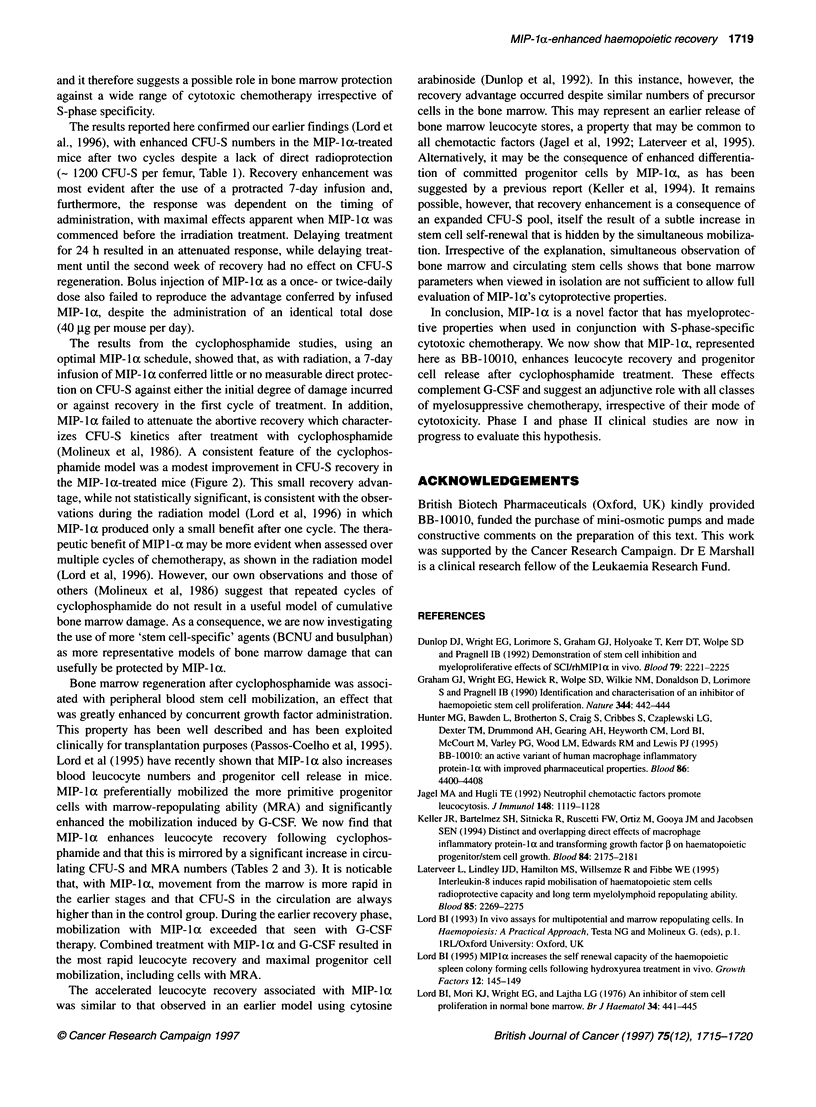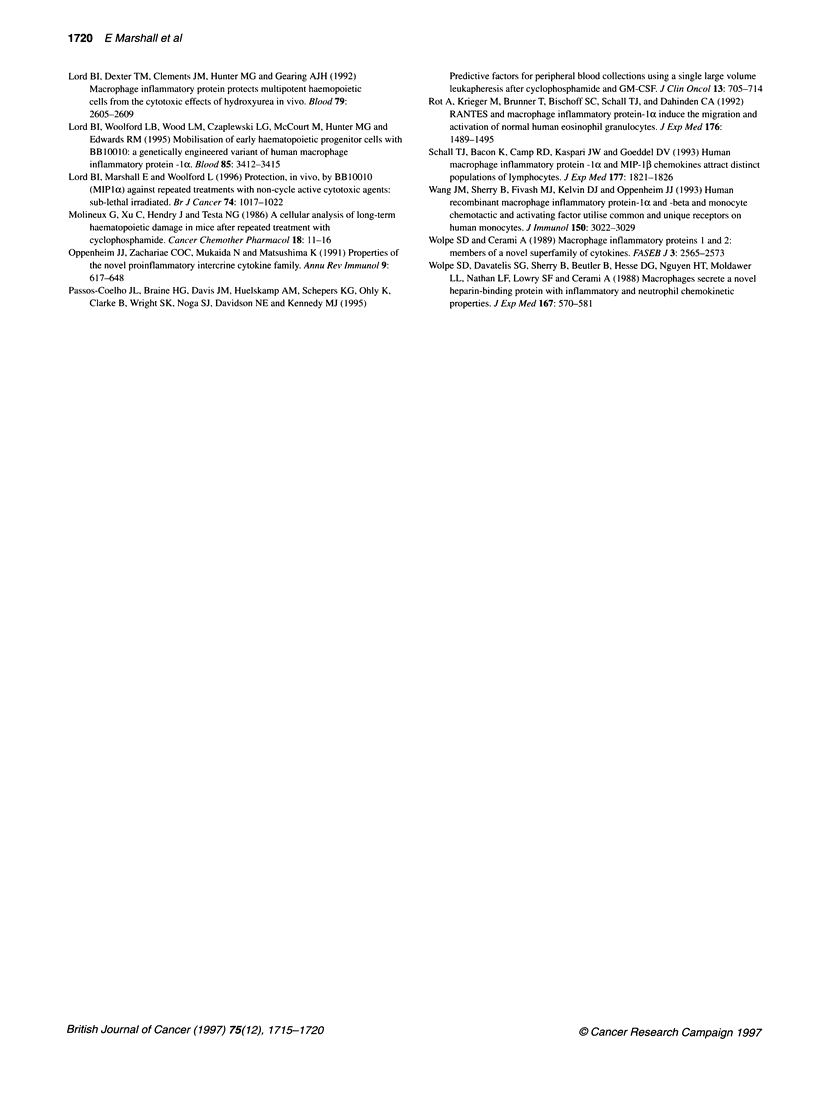# Continuous infusion of macrophage inflammatory protein MIP-1alpha enhances leucocyte recovery and haemopoietic progenitor cell mobilization after cyclophosphamide.

**DOI:** 10.1038/bjc.1997.294

**Published:** 1997

**Authors:** E. Marshall, L. B. Woolford, B. I. Lord

**Affiliations:** CRC Department of Medical Oncology, Christie Hospital, Manchester, UK.

## Abstract

Macrophage inflammatory protein 1alpha (MIP-1alpha) inhibits haemopoietic stem cell proliferation. This property has been exploited in a murine chemotherapy model and has been shown to ameliorate cytotoxic-induced myelosuppression after S-phase-specific cytotoxic therapy. We have now shown that BB-10010, a stable mutant of MIP-1alpha, (a) is more effective when administered as a continuous infusion than when bolus injected and (b), when administered via a 7-day infusion during and after cyclophosphamide treatment, results in an earlier recovery of leucocyte numbers. This effect was accompanied by progenitor cell mobilization into the peripheral blood and included primitive cells with marrow-repopulating ability (MRA). Maximal mobilization and recovery of leucocytes occurred when MIP-1alpha was combined with granulocyte colony-stimulating factor (G-CSF) therapy. The findings suggest that MIP1-alpha used alone or in combination with G-CSF may allow delivery of a greater chemotherapy dose intensity as a consequence of both accelerated leucocyte recovery and maintenance of high-quality mobilized progenitor cells for harvesting and peripheral blood stem cell transplantation.


British Journal of Cancer (1997) 75(12), 1715–1720
© 1997 Cancer Research Campaign

### Continuous infusion of macrophage inflammatory protein MIP-1α enhances leucocyte recovery and haemopoietic progenitor cell mobilization after cyclophosphamide

**E Marshall[1]*, LB Woolford[2] and BI Lord[2]**

[1]CRC Department of Medical Oncology, Christie Hospital, Manchester M20 4BX, UK; [2]CRC Department of Experimental Haematology, Paterson Institute for Cancer Research, Manchester M20 4BX, UK

**Summary** Macrophage inflammatory protein 1α (MIP-1α) inhibits haemopoietic stem cell proliferation. This property has been exploited in a murine chemotherapy model and has been shown to ameliorate cytotoxic-induced myelosuppression after S-phase-specific cytotoxic therapy. We have now shown that BB-10010, a stable mutant of MIP-1α, (a) is more effective when administered as a continuous infusion than when bolus injected and (b), when administered via a 7-day infusion during and after cyclophosphamide treatment, results in an earlier recovery of leucocyte numbers. This effect was accompanied by progenitor cell mobilization into the peripheral blood and included primitive cells with marrow-repopulating ability (MRA). Maximal mobilization and recovery of leucocytes occurred when MIP-1α was combined with granulocyte colony-stimulating factor (G-CSF) therapy. The findings suggest that MIP1-α used alone or in combination with G-CSF may allow delivery of a greater chemotherapy dose intensity as a consequence of both accelerated leucocyte recovery and maintenance of high-quality mobilized progenitor cells for harvesting and peripheral blood stem cell transplantation.

**Keywords:** MIP-1α; BB-10010; mobilization; granulocyte colony-stimulating factor; myeloprotection

Macrophage inflammatory protein 1α (MIP-1α) is an 8-kDa basic heparin-binding polypeptide that possesses proinflammatory and reparative activity (Wolpe et al, 1988; Oppenheim et al, 1991). It is defined as a c-c chemokine on the basis of functional and structural similarities with other family members, including monocyte chemotactic activating factor (MCAF) and RANTES (Wolpe et al, 1989; Oppenheim et al, 1991). As a component of the inflammatory response, MIP-1α is chemotactic for selected leucocyte subsets (Wolpe et al, 1989; Oppenheim et al, 1991; Rot et al, 1992; Schall et al, 1993; Wang et al, 1993). Recently, MIP-1α was described (Graham et al, 1990) as the active component of a normal bone marrow extract (NBME-IV; Lord et al, 1976), which inhibits the proliferation of multipotential haemopoietic progenitor cells – stem cells assayed as spleen colony-forming units (CFU-S). The potential to inhibit stem cell entry into DNA synthesis provides a novel therapeutic strategy for protecting normal bone marrow from the cytotoxic effects of chemotherapeutic agents. This was confirmed following the administration of the S-phase-specific cytotoxic drugs, hydroxyurea (HU) (Lord et al, 1992) and cytosine arabinoside (Ara C) (Dunlop et al, 1992). In both models, MIP-1α, administered around the timing of chemotherapy, ameliorated stem cell loss and resulted in an earlier and more rapid recovery of the stem cell population. Furthermore, protection of the stem cell compartment was reflected by a significant improvement in the kinetics of neutrophil recovery (Dunlop et al, 1992). It now appears that MIP-1α possesses additional properties to that of simple cell cycle inhibition. Analysis of stem cell (CFU-S) recovery following HU and MIP-1α suggests that MIP-1α may also modulate the self-renewal and differentiation capacity of the stem cell population (Lord, 1995). Self-renewal of CFU-S during the recovery period was 50% higher than during untreated recovery. The resultant increase in the stem cell pool was then more than sufficient to offset the complementary reduction in differentiation rate and thus allowed the more rapid neutrophil recovery. This, together with the recent report that MIP-1α mobilizes haemopoietic progenitor cells (Lord et al, 1995), suggests that MIP-1α may offer further therapeutic benefit when used in conjunction with the more clinically relevant, non-S-phase-specific cytotoxic agents.

Evaluation of MIP-1α has been hindered by a tendency of the native protein to undergo aggregation and form high molecular weight polymers. BB-10010 represents a stable mutant carrying a single amino acid substitution of Asp26 ⇒ Ala with a reduced tendency to form polymers at physiological pH and ionic strength (Hunter et al, 1996). The potency of BB-10010 appears to be similar to native MIP-1α, as judged by receptor binding, calcium mobilization, thymidine suicide and murine myeloprotective experiments (Hunter et al, 1996).

Here, we investigate the efficacy of continuous infusion of MIP-1α (as represented by BB-10010) compared with repeated bolus injection and demonstrate the beneficial effects of MIP-1α on the recovery kinetics of bone marrow in a murine model of cyclophosphamide-induced bone marrow damage.

*Received 26 June 1996*
*Revised 4 November 1996*
*Accepted 3 January 1997*

*Correspondence to:* E Marshall, Department of Medical Oncology, Clatterbridge Centre for Oncology, Bebington, Wirral L63 4JY, UK

**Present address:* Clatterbridge Centre for Oncology, Bebington, Wirral L63 4JY, UK

### MATERIALS AND METHODS

### Animals

Female B6D2F1(C57B1 female × DBA2 male) mice aged 10 weeks were used throughout and all procedures were carried out, under licence, according to the provisions of the Home Office Animals (Scientific Procedures) Act, 1986.

### Injection of cyclophosphamide

Cyclophosphamide powder (farmtalia Carlo Erba) was dissolved in isotonic saline. A final dose of 200 mg $kg^{-1}$ was injected intraperitoneally (i.p.) into preweighed mice.

### Cytokines

MIP-1α was kindly supplied by British Biotech Pharmaceuticals (Oxford, UK) as a non-aggregating genetically engineered variant of human MIP-1α (LD78); it is currently known as BB-10010. It was administered either by subcutaneous injection or by mini-osmotic pumps (Alzet 2001, CA, USA) implanted subcutaneously on the backs of mice and delivered at a constant infusion rate of 40 μg per mouse $day^{-1}$ for 3 or 7 days. Recombinant human G-CSF (Amgen, Thousand Oakes) was injected at a dose of 100 μg/$kg^{-1}$ subcutaneously every 12 h from day 3 to day 7.

### Preparation of cell suspensions

Blood was collected by terminal cardiac puncture under light anaesthesia (ethrane) and pooled from the various groups of donor mice. Heparin (25 μ $ml^{-1}$) was used as an anticoagulant. Leucocyte counts were performed on an automated counter (Sysmex). Bone marrow cells were harvested by flushing the femur with Fischer's medium using a 21G needle as previously described (Lord, 1993).

### Colony assays

Eight- and 12-day CFU-S (CFU-$S_8$, CFU-$S_{12}$) and cells with marrow-repopulating ability (MRA) were assayed as described in detail previously (Lord, 1993). Briefly, mice (groups of 20) were exposed to 15.25-Gy $^{60}$Co γ-ray irradiation at 0.85 Gy $h^{-1}$. They were then injected with a freshly prepared suspension of bone marrow or whole blood. For this, known fractions (1:50–1:400) of donor femoral marrow were adjusted to generate approximately 10 colonies per spleen from an injection volume of 0.2 ml. Similarly, venesected blood (5–100 μl) was diluted to a final volume of 0.2 ml, again to generate approximately ten colonies per spleen in recipient mice. Eight and 12 days later, ten recipient mice were killed. Their spleens were fixed and the colonies counted. Cells with MRA were measured by transplanting haemopoietic tissue from the donor into an additional five primary irradiated recipient mice. the transplanted cells were allowed to engraft and develop in the marrow for 13 days before harvesting and transplanting into a secondary group of 10 irradiated recipient mice for a CFU-$S_{12}$ assay. Results are expressed as CFU-S per femur ± s.e., CFU-S per ml of whole blood ± s.e. or $MRA^{-1}$ ml± s.e. of whole blood.

### Experimental protocols

Two separate experimental protocols were designed to determine (a) the optimal schedule of MIP-1α delivery using a previously described model of repeated sublethal irradiation (Lord, 1996) and (b) to investigate the myeloprotective and mobilization properties of MIP-1α against cyclophosphamide.

#### *MIP-1α scheduling*

Groups of three mice were irradiated with 4.5-Gy whole-body γ-rays. They were administered MIP-1α or placebo (phosphate-buffered saline) either by subcutaneous injection for 7 days or subcutaneous infusion using an inplanted 7-day mini-osmotic pump (Alzet 2001). Pumps were inserted under short-acting anaesthetic (ethrane) at variable time points (3–4 h before or 24 h or 7 days after irradiation) and removed 3 or 7 days later. After 14 days, this cycle of radiation and treatment was repeated. In all experiments, groups of three mice were killed at day 14 of treatment cycle 2 and their femoral marrow assayed, in this experiment, for CFU-$S_{10}$.

#### *Myeloprotection against cyclophosphamide and mobilization of progenitor cells*

Groups of three mice received a single i.p. injection of cyclophosphamide and the appropriate treatment with MIP-1α, G-CSF or both. MIP-1α was administered for 7 days using a mini-pump

**Table 1** Recovery of bone marrow CFU-S after two cycles of sublethal irradiation using a variable MIP-1α schedule

| Treatment | CFU-S per femur | | |
|---|---|---|---|
| | Study I | Study II | Study III |
| 4.5-Gy γ-rays | 458 ± 51 | 554 ± 58 | 730 ± 94 |
| 7-day MIP-1α (0–7)* | 1250 ± 108 | 1220 ± 77 | 1270 ± 127 |
| 7-day MIP-1α (1–8) | – | – | 960 ± 69 |
| 7-day MIP-1α (7–14) | – | – | 690 ± 64 |
| 3-day MIP-1α (0–3) | 799 ± 81** | – | – |
| Daily bolus MIP-1α | – | 711 ± 67 | – |
| Twice-daily bolus MIP-1α | – | 550 ± 56 | – |
| Number of experiments | 3 | 3 | 3 |

The results show the means ± s.e. of three experiments (a total of nine experiments in all) for day 10 CFU-S. In all experiments MIP-1α was administered at a dose of 40 μg per mouse per day. *$P$ < 0.001 (significance level for the combined MIP-1α-treated studies vs combined controls); **$P$ < 0.05.

**Figure 1** Bone marrow cellularity after cyclophosphamide with and without MIP-1α. Results are the means ± s.e. of four experiments. *$P$ = 0.05. -◆-, cyclophosphamide; -■-, cyclophosphamide + MIP-1α

© Cancer Research Campaign 1997

**Figure 2** (**A**) CFU-$S_8$ and (**B**) CFU-$S_{12}$ per femur after cyclophosphamide with and without MIP-1α. Results are the means ± s.e. of three experiments. *$P < 0.001$. -◆-, cyclophosphamide; -■-, cyclophosphamide + MIP-1α

inserted under short-acting anaesthetic, approximately 3 h before cytotoxic treatment. G-CSF (100 μg $kg^{-1}$) was injected subcutaneously twice daily from day 3 to day 7. Control mice received cyclophosphamide only. Femoral bone marrow cellularity and CFU-S were assayed at time points between days 1 and 14. Peripheral white blood cell (WBC) counts were made daily from day 1 to day 10, and the mobilized progenitor cells were assayed daily from day 4 to day 7 after cyclophosphamide treatment.

### Statistical analysis

For each experiment, the means of the respective haematological parameters were calculated and expressed as means ± standard error plotted against time. When appropriate, results were analysed using a two-sided Student's *t*-test.

**Figure 3** Leucocyte recovery after cyclophosphamide with and without MIP-1α. Results are the mean ± s.e. of three experiments. *$P < 0.01$. -◆-, cyclophosphamide; -■-, cyclophosphamide + MIP-1α

### RESULTS

### MIP-1α scheduling and irradiation

*Effect of continuous infusion of MIP-1α on CFU-S recovery following irradiation*

Table 1 shows the results of three separate studies comparing the effects of timing and bolus vs continuous administration of MIP-1α. Two cycles of 4.5-Gy γ-ray irradiation reduced the femoral CFU-$S_{10}$ to approximately 10% of normal (458 ± 51) at day 14. The continuous administration of MIP-1α increased the CFU-S recovery to 1250 ($P < 0.001$) and 800 CFU-S per femur ($P < 0.05$) after 7-day and 3-day infusions respectively (study I, Table 1). This effect of MIP-1α was lost when the same total dose was administered as a daily (711 CFU-S per femur, $P > 0.1$) or twice daily (550 CFU-S per femur, $P > 0.1$) bolus injection (study II, Table 1). Finally, the response to MIP-1α was dependent on the timing of administration relative to the myelosuppressive insult (study III, Table 1). Commencement of 7-day continuous MIP-1α treatment immediately preceding irradiation conferred maximal advantage. An attenuated effect (960 ± 69) was evident when the MIP-1α infusion was commenced 24 h after irradiation and was totally abrogated when administered during the second half of each cycle (690 ± 64).

### Myeloprotection against cyclophosphamide and mobilization of progenitor cells

*Bone marrow recovery*

Bone marrow cellularity (Figure 1) showed a similar degree of suppression at day 1, following cyclophosphamide, irrespective of additional MIP-1α treatment. MIP-1α did however generate an

**Table 2** Total leucocyte count ($10^9$ $l^{-1}$) after cyclophosphamide treatment with and without MIP-1α and/or G-CSF

| Treatment | Day 4 | Day 5 | Day 6 | Day 7 |
|---|---|---|---|---|
| Cyclophosphamide | 2 ± 0.4 | 1.6 ± 0.1 | 3.1 ± 0.4 | 7.3 ± 0.1 |
| Cyclophosphamide + MIP-1α | 2 ± 0.5 | 2.7 ± 0.6 | 6.6 ± 0.3** | 14.2 ± 4.0 |
| Cyclophosphamide + G-CSF | 1.5 ± 0.2 | 5.5 ± 1.1* | 28.4 ± 1.6*** | 26.4 ± 2.5** |
| Cyclophosphamide + MIP-1α + G-CSF | 2.3 ± 0.4 | 14.8 ± 4.7* | 26.2 ± 4.9** | 26.5 ± 2.3** |

Results show the mean ± s.e. of three experiments. *$P < 0.05$; **$P < 0.01$; ***$P < 0.001$.

© Cancer Research Campaign 1997 

**Figure 4** Comparison of bone marrow (BM) and blood CFU-$S_8$ after cyclophosphamide. Results are the means of three experiments. -◆-. cyclophosphamide (blood); -■-, cyclophosphamide + MIP-1α (blood); -▲-, cyclophosphamide (BM); -×-, cyclophosphamide + MIP-1α (BM)

acceleration of recovery at day 4 (12.7 ± 2.9 × $10^6$ per femur vs 5.8 ± 0.6 × $10^6$ per femur, $P = 0.05$). Recovery to control numbers was complete in both groups by day 8.

The recovery patterns of the day 8 CFU-S and day 12 CFU-S populations are shown in Figure 2. Cyclophosphamide induced a rapid fall in both CFU-S populations with a nadir occurring at day 1. By 14 days, both CFU-$S_8$ and CFU-$S_{12}$ were approaching their normal numbers (approximately 3500 per femur) having demonstrated an abortive recovery phase by around day 4. The 8-day CFU-S were more sensitive to the cytotoxic effects of cyclophosphamide when comparing day 1 survivals in both populations (CFU-$S_8$ 217 ± 37 per femur vs CFU-$S_{12}$ 930 ± 70 per femur, $P < 0.001$). Co-administration of MIP-1α neither provided any measurable protection to the cells from their initial depletion nor enhanced their recovery in the first 8 days. Although the MIP-1α-treated groups had consistently higher day 8 and day 12 CFU-S numbers during the later regeneration period, the difference was small and fell within one standard error of the means by day 14.

### *Leucocyte recovery*

The total leucocyte counts over 10 days after cyclophosphamide treatment are shown in Figure 3. For the first 3 days, the WBC remained in the normal range after which cyclophosphamide treatment produced a short-lasting leucopenia (days 4–6) with a mean nadir leucocyte count of 1.6 ± 0.1 × $10^9$ $l^{-1}$. Mice receiving concurrent MIP-1α developed a similar nadir (2.0 ± 0.5 × $10^9$ $l^{-1}$) but recovered to control leucocyte numbers 1 day earlier (day 6) ($P < 0.01$) and overshot twofold by day 7. Leucocyte recovery was enhanced by the addition of either MIP-1α or G-CSF treatment (Table 2). On its own, MIP-1α resulted in only a modest twofold improvement in the rate of leucocyte recovery compared with control – normal levels were reached by day 6 compared with day 7 and overshooting to 14.2 × $10^9$ leucocytes $l^{-1}$ on day 7 (Figure 3). By comparison, G-CSF normalized leucocyte numbers by day 5 but combined MIP-1α and G-CSF accelerated recovery even further, giving a considerable overshoot of 14.8 ± 4.7 $l^{-1}$ by day 5.

The changes in bone marrow progenitor cell numbers during mobilization are shown in Figure 4. CFU-S numbers fell off acutely, after their initial abortive recovery at day 4, to a second nadir which corresponded to the increasing mobilization of progenitor cells into the peripheral blood. The enhanced mobilization seen with MIP-1α was reflected by a more rapid reduction in day 8 CFU-S between day 4 and day 6. At day 7, femoral CFU-S numbers were similar in both groups (cyclophosphamide 630 ± 40 vs MIP-1α 450 ± 50, $P > 0.05$) despite a 2.4-fold increase in mobilized CFU-S in the MIP-1α-treated cohort.

### DISCUSSION

MIP-1α has been shown to protect multipotential haemopoietic progenitor cells against repeated treatments with S-phase chemotherapeutic drugs (Dunlop et al, 1992; Lord et al, 1992), however it remains to be seen whether a similar stratagem will alleviate the myelosuppressive effects of the more clinically relevant, non-S-phase-specific anti-cancer agents.

We have devised a 7-day schedule of continuous MIP-1α (40 μg per mouse $day^{-1}$) administered via an implanted subcutaneous pump and inserted before chemotherapy. This dosing schedule was based on extensive preclinical studies with MIP-1α, including a murine model of MIP-1α-induced radioprotection (Lord et al, 1996). In this model, MIP-1α attenuated the incremental bone marrow damage associated with repeated treatments with sublethal irradiation (450 rads γ-rays every 14 days for four cycles; Lord et al, 1996). No direct myeloprotection was observed but the cumulative effects of the enhanced recovery of CFU-Ss became most notable during the later cycles of treatment. Mechanistically, the observed response to MIP-1α was felt to be most consistent with an improved self-renewal capability of the surviving CFU-Ss,

**Table 3** CFU-S $ml^{-1}$ blood after cyclophosphamide treatment with and without MIP-1α and/or G-CSF

| Treatment | Day 4 | Day 5 | Day 6 | Day 7 |
|---|---|---|---|---|
| *CFU-S per ml of blood* | | | | |
| Cyclophosphamide | 14 ± 6 | 217 ± 63 | 462 ± 84 | 612 ± 140 |
| Cyclophosphamide + MIP-1α | 318 ± 58[2] | 572 ± 129 | 795 ± 189 | 1430 ± 542 |
| Cyclophosphamide + G-CSF | 220 ± 111 | 528 ± 108 | 1911 ± 678 | 2256 ± 374[3] |
| Cyclophosphamide + MIP-1α + G-CSF | 504 ± 47[4] | 1032 ± 221[1] | 2303 ± 720[1] | 2371 ± 545[1] |
| *MRA per ml of blood* | | | | |
| Cyclophosphamide | 1499 ± 250 | 2749 ± 900 | 3925 ± 175 | 5210 ± 1650 |
| Cyclophosphamide + MIP-1α | 8491 ± 1490[2] | 16600 ± 610[4] | 21450 ± 1390[4] | 26500 ± 1500[4] |
| Cyclophosphamide + G-CSF | 6493 ± 333[4] | 11300 ± 1340[2] | 17500 ± 3000[3] | 24500 ± 2500[2] |
| Cyclophosphamide + MIP-1α + G-CSF | 9540 ± 1715[1] | 28440 ± 2870[2] | 32000 ± 3200[4] | 45000 ± 5000[2] |

Results show the means ± s.e. of three experiments. [1]$P < 0.05$, [2]$P < 0.01$, [3]$P < 0.02$, [4] < 0.001.

© Cancer Research Campaign 1997

and it therefore suggests a possible role in bone marrow protection against a wide range of cytotoxic chemotherapy irrespective of S-phase specificity.

The results reported here confirmed our earlier findings (Lord et al., 1996), with enhanced CFU-S numbers in the MIP-1α-treated mice after two cycles despite a lack of direct radioprotection (~ 1200 CFU-S per femur, Table 1). Recovery enhancement was most evident after the use of a protracted 7-day infusion and, furthermore, the response was dependent on the timing of administration, with maximal effects apparent when MIP-1α was commenced before the irradiation treatment. Delaying treatment for 24 h resulted in an attenuated response, while delaying treatment until the second week of recovery had no effect on CFU-S regeneration. Bolus injection of MIP-1α as a once- or twice-daily dose also failed to reproduce the advantage conferred by infused MIP-1α, despite the administration of an identical total dose (40 μg per mouse per day).

The results from the cyclophosphamide studies, using an optimal MIP-1α schedule, showed that, as with radiation, a 7-day infusion of MIP-1α conferred little or no measurable direct protection on CFU-S against either the initial degree of damage incurred or against recovery in the first cycle of treatment. In addition, MIP-1α failed to attenuate the abortive recovery which characterizes CFU-S kinetics after treatment with cyclophosphamide (Molineux et al, 1986). A consistent feature of the cyclophosphamide model was a modest improvement in CFU-S recovery in the MIP-1α-treated mice (Figure 2). This small recovery advantage, while not statistically significant, is consistent with the observations during the radiation model (Lord et al, 1996) in which MIP-1α produced only a small benefit after one cycle. The therapeutic benefit of MIP1-α may be more evident when assessed over multiple cycles of chemotherapy, as shown in the radiation model (Lord et al, 1996). However, our own observations and those of others (Molineux et al, 1986) suggest that repeated cycles of cyclophosphamide do not result in a useful model of cumulative bone marrow damage. As a consequence, we are now investigating the use of more 'stem cell-specific' agents (BCNU and busulphan) as more representative models of bone marrow damage that can usefully be protected by MIP-1α.

Bone marrow regeneration after cyclophosphamide was associated with peripheral blood stem cell mobilization, an effect that was greatly enhanced by concurrent growth factor administration. This property has been well described and has been exploited clinically for transplantation purposes (Passos-Coelho et al, 1995). Lord et al (1995) have recently shown that MIP-1α also increases blood leucocyte numbers and progenitor cell release in mice. MIP-1α preferentially mobilized the more primitive progenitor cells with marrow-repopulating ability (MRA) and significantly enhanced the mobilization induced by G-CSF. We now find that MIP-1α enhances leucocyte recovery following cyclophosphamide and that this is mirrored by a significant increase in circulating CFU-S and MRA numbers (Tables 2 and 3). It is noticable that, with MIP-1α, movement from the marrow is more rapid in the earlier stages and that CFU-S in the circulation are always higher than in the control group. During the earlier recovery phase, mobilization with MIP-1α exceeded that seen with G-CSF therapy. Combined treatment with MIP-1α and G-CSF resulted in the most rapid leucocyte recovery and maximal progenitor cell mobilization, including cells with MRA.

The accelerated leucocyte recovery associated with MIP-1α was similar to that observed in an earlier model using cytosine arabinoside (Dunlop et al, 1992). In this instance, however, the recovery advantage occurred despite similar numbers of precursor cells in the bone marrow. This may represent an earlier release of bone marrow leucocyte stores, a property that may be common to all chemotactic factors (Jagel et al, 1992; Laterveer et al, 1995). Alternatively, it may be the consequence of enhanced differentiation of committed progenitor cells by MIP-1α, as has been suggested by a previous report (Keller et al, 1994). It remains possible, however, that recovery enhancement is a consequence of an expanded CFU-S pool, itself the result of a subtle increase in stem cell self-renewal that is hidden by the simultaneous mobilization. Irrespective of the explanation, simultaneous observation of bone marrow and circulating stem cells shows that bone marrow parameters when viewed in isolation are not sufficient to allow full evaluation of MIP-1α's cytoprotective properties.

In conclusion, MIP-1α is a novel factor that has myeloprotective properties when used in conjunction with S-phase-specific cytotoxic chemotherapy. We now show that MIP-1α, represented here as BB-10010, enhances leucocyte recovery and progenitor cell release after cyclophosphamide treatment. These effects complement G-CSF and suggest an adjunctive role with all classes of myelosuppressive chemotherapy, irrespective of their mode of cytotoxicity. Phase I and phase II clinical studies are now in progress to evaluate this hypothesis.

### ACKNOWLEDGEMENTS

British Biotech Pharmaceuticals (Oxford, UK) kindly provided BB-10010, funded the purchase of mini-osmotic pumps and made constructive comments on the preparation of this text. This work was supported by the Cancer Research Campaign. Dr E Marshall is a clinical research fellow of the Leukaemia Research Fund.

### REFERENCES

Dunlop DJ, Wright EG, Lorimore S, Graham GJ, Holyoake T, Kerr DT, Wolpe SD and Pragnell IB (1992) Demonstration of stem cell inhibition and myeloproliferative effects of SCI/rhMIP1α in vivo. *Blood* **79**: 2221–2225

Graham GJ, Wright EG, Hewick R, Wolpe SD, Wilkie NM, Donaldson D, Lorimore S and Pragnell IB (1990) Identification and characterisation of an inhibitor of haemopoietic stem cell proliferation. *Nature* **344**: 442–444

Hunter MG, Bawden L, Brotherton S, Craig S, Cribbes S, Czaplewski LG, Dexter TM, Drummond AH, Gearing AH, Heyworth CM, Lord BI, McCourt M, Varley PG, Wood LM, Edwards RM and Lewis PJ (1995) BB-10010: an active variant of human macrophage inflammatory protein-1α with improved pharmaceutical properties. *Blood* **86**: 4400–4408

Jagel MA and Hugli TE (1992) Neutrophil chemotactic factors promote leucocytosis. *J Immunol* **148**: 1119–1128

Keller JR, Bartelmez SH, Sitnicka R, Ruscetti FW, Ortiz M, Gooya JM and Jacobsen SEN (1994) Distinct and overlapping direct effects of macrophage inflammatory protein-1α and transforming growth factor β on haematopoietic progenitor/stem cell growth. *Blood* **84**: 2175–2181

Laterveer L, Lindley IJD, Hamilton MS, Willsemze R and Fibbe WE (1995) Interleukin-8 induces rapid mobilisation of haematopoietic stem cells radioprotective capacity and long term myelolymphoid repopulating ability. *Blood* **85**: 2269–2275

Lord BI (1993) In vivo assays for multipotential and marrow repopulating cells. In *Haemopoiesis: A Practical Approach,* Testa NG and Molineux G. (eds), p.1. IRL/Oxford University: Oxford, UK

Lord BI (1995) MIP1α increases the self renewal capacity of the haemopoietic spleen colony forming cells following hydroxyurea treatment in vivo. *Growth Factors* **12**: 145–149

Lord BI, Mori KJ, Wright EG, and Lajtha LG (1976) An inhibitor of stem cell proliferation in normal bone marrow. *Br J Haematol* **34**: 441–445

© Cancer Research Campaign 1997 

Lord BI, Dexter TM, Clements JM, Hunter MG and Gearing AJH (1992) Macrophage inflammatory protein protects multipotent haemopoietic cells from the cytotoxic effects of hydroxyurea in vivo. *Blood* **79**: 2605–2609

Lord BI, Woolford LB, Wood LM, Czaplewski LG, McCourt M, Hunter MG and Edwards RM (1995) Mobilisation of early haematopoietic progenitor cells with BB10010: a genetically engineered variant of human macrophage inflammatory protein -1α. *Blood* **85**: 3412–3415

Lord BI, Marshall E and Woolford L (1996) Protection, in vivo, by BB10010 (MIP1α) against repeated treatments with non-cycle active cytotoxic agents: sub-lethal irradiated. *Br J Cancer* **74**: 1017–1022

Molineux G, Xu C, Hendry J and Testa NG (1986) A cellular analysis of long-term haematopoietic damage in mice after repeated treatment with cyclophosphamide. *Cancer Chemother Pharmacol* **18**: 11–16

Oppenheim JJ, Zachariae COC, Mukaida N and Matsushima K (1991) Properties of the novel proinflammatory intercrine cytokine family. *Annu Rev Immunol* **9**: 617–648

Passos-Coelho JL, Braine HG, Davis JM, Huelskamp AM, Schepers KG, Ohly K, Clarke B, Wright SK, Noga SJ, Davidson NE and Kennedy MJ (1995) Predictive factors for peripheral blood collections using a single large volume leukapheresis after cyclophosphamide and GM-CSF. *J Clin Oncol* **13**: 705–714

Rot A, Krieger M, Brunner T, Bischoff SC, Schall TJ, and Dahinden CA (1992) RANTES and macrophage inflammatory protein-1α induce the migration and activation of normal human eosinophil granulocytes. *J Exp Med* **176**: 1489–1495

Schall TJ, Bacon K, Camp RD, Kaspari JW and Goeddel DV (1993) Human macrophage inflammatory protein -1α and MIP-1β chemokines attract distinct populations of lymphocytes. *J Exp Med* **177**: 1821–1826

Wang JM, Sherry B, Fivash MJ, Kelvin DJ and Oppenheim JJ (1993) Human recombinant macrophage inflammatory protein-1α and -beta and monocyte chemotactic and activating factor utilise common and unique receptors on human monocytes. *J Immunol* **150**: 3022–3029

Wolpe SD and Cerami A (1989) Macrophage inflammatory proteins 1 and 2: members of a novel superfamily of cytokines. *FASEB J* **3**: 2565–2573

Wolpe SD, Davatelis SG, Sherry B, Beutler B, Hesse DG, Nguyen HT, Moldawer LL, Nathan LF, Lowry SF and Cerami A (1988) Macrophages secrete a novel heparin-binding protein with inflammatory and neutrophil chemokinetic properties. *J Exp Med* **167**: 570–581

*© Cancer Research Campaign 1997*